# Effective Dehydration of Fructose Over Stable Ti-Doped SBA-15 Catalysts

**DOI:** 10.3389/fchem.2021.817417

**Published:** 2022-01-05

**Authors:** Yutong Zhu, Xiaofei Xu, Jian He, Jie Guo, Ke Song

**Affiliations:** ^1^ Key Laboratory of Hunan Forest Products and Chemical Industry Engineering, Jishou University, Zhangjiajie, China; ^2^ College of Chemistry and Chemical Engineering, Jishou University, Jishou, China

**Keywords:** 5-hydroxymethylfurfural, fructose, SBA-15, dehydration, heterogeneous catalysis

## Abstract

High-effective synthesis of 5-hydroxymethylfurfural (HMF) from carbohydrates is an interesting reaction among biomass valorization. The as-synthesized Ti-SBA-15 catalysts with mesoporous structures showed high catalytic efficiency for the conversion of fructose to HMF. Ti-SBA-15 catalysts with different Si/Ti ratios were characterized by characterization techniques such as elemental analysis, XRD, TEM, N_2_ adsorption–desorption, NH_3_-TPD, and pyridine-FTIR. The acidity of Ti-SBA-15 catalysts could be tuned by altering addition amount of titanium. The effects of reaction conditions, including reaction time, temperature, and amount of catalyst, on the conversions of fructose and the yields of HMF were also investigated. It is found that Ti-SBA-15 catalysts whose Si/Ti ratio is 120 gave the best yields of HMF, which demonstrated 100% conversion of fructose with a maximum HMF yield of 82% at 140°C after 1 h. In addition, its catalytic performance was retained after 5 recycles in fructose conversion reaction, proving its good catalytic stability.

## Introduction

**FIGURE 1:**
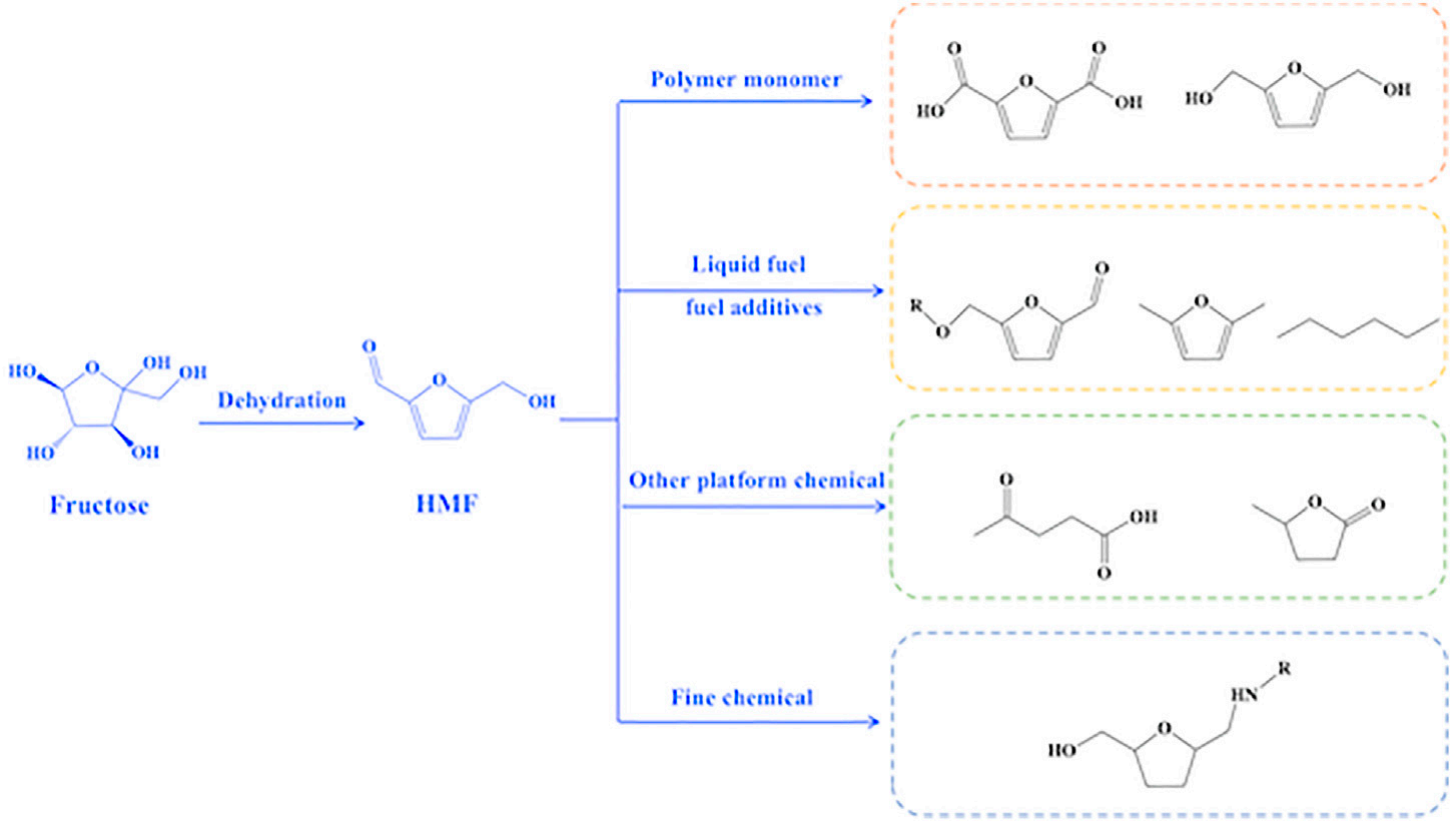
Reaction pathway of dehydration of fructose into HMF and illustrations for HMF application.

Petroleum-based energy shortage and environmental pollution have become global issues due to over-reliance on finite fossil fuels, which caused considerable search for alternatives to the limited fossil resources to efficiently supply of energy and chemicals (Yue et al., 2016). Biomass, a renewable organic carbon sources, has great potential to substitute fossil re-sources for the production of fuels and chemicals because it is abundant and easily available. The conversion of biomass into energy and chemicals generally begins with the conversion of biomass into platform molecules. Consequently, various bio-based molecules (e.g., HMF, lactic acid, 2,5-furandicarboxylic acid, and levulinic acid) are yielded from biomass, which serve as important starting feedstocks for synthesis of fuels and chemicals (Bozell and Petersen, 2010; Sudarsanam et al., 2018; Jing et al., 2019; Ma et al., 2020). HMF has attracted researchers’ interest as a prospective biorefinery intermediate and precursor for medicines, polymer monomers, and fuels. Because of its furan ring structure with one aldehyde and one alcohol group, HMF is regarded as a major platform compound among biomass-derived compounds. The special chemical structure character of HMF makes it very active (Figure 1) to be converted into a series of compounds including polymer precursors [2,5-furan dicarboxylic acid and bis-2,5-(hydroxymethyl)furan], liquid fuel precursor, and fuel additives (5-acetoxymethyl-2-furaldehyde, N,N-dimethylformamide, and n-hexane) as well as other platform chemicals (levulinic acid and γ-valerolactone).

Currently, in the studies of HMF production, fructose, glucose, and cellulose are often used as the feedstocks (Tzeng et al., 2019). Complex carbohydrates, such as cellulose, can be transformed to HMF in three steps: cellulose hydrolysis to glucose, glucose isomerization to fructose, and fructose dehydration to HMF. The final step of these carbohydrates conversion into HMF is the dehydration of fructose to HMF. Thus, high-effective synthesis of HMF from fructose will lay the foundation for biomass-to-HMF transformation (Jiménez-Morales et al., 2014). Actually, fructose is considered as the most efficient and common raw material for HMF synthesis, which allows higher yields of HMF to be obtained (Zhang et al., 2017) because its fructofuranoic structure is more reactive to dehydration (Wang et al., 2019). Generally, dehydration of fructose into HMF is an acid-catalyzed reaction and many homogeneous acid catalysts, such as the easily available mineral acids [H_2_SO_4_ (Zuo et al., 2017), HCl (Zuo et al., 2017), H_3_PO_4_ (Ray et al., 2011), CH_3_COOH (Thananatthanachon and Rauchfuss, 2010), etc.] and salts [AlCl_3_ (Zuo et al., 2017), CrCl_3_ (Yong et al., 2008), ZnCl_2_ (Zuo et al., 2017), etc.], have been studied in dehydration of fructose to produce HMF. The above mentioned homogeneous catalytic systems, however, usually suffer from corrosivity and difficulty in catalysts separation and reuse (Zhang et al., 2020).

Recently, various heterogeneous catalysts such as metal oxides (DaVià et al., 2017), zeolites (Nie et al., 2020), resins (Qi et al., 2009; Atanda et al., 2015), metal phosphates (Dibenedetto et al., 2016), and heteropoly acids (Gomes et al., 2017) have been successfully developed for dehydration of fructose into HMF, in order to avert the issues associated with using homogeneous catalysts. Although excellent results are obtained, those catalysts encounter some issues including small specific surface area [e.g., resin, S_BET_ < 50 m^2^/g), non-adjustable pore structure, and heteropoly acids (such as H_3_PW_12_O_40_ (Gomes et al., 2017)] dissolved to some extent in DMSO and low active (e.g., metal oxide catalysts). As a result, it is critical to develop an effective, low-cost, and hydrothermally stable catalyst for conversion of carbohydrate to HMF.

SBA-15 mesoporous silica materials, featured with high surface area, relatively large and tunable pore size, as well as facile surface functionalization properties, have been widely applied in catalysis fields (Wang et al., 2019). Another feature of this material is that the mesoporous channels are linked by microporous channels (Wróblewska et al., 2018). It has superior hydrothermal properties and acid resistance when compared to other materials in this group, such as MCM-41. The unique molecular structure and mesoporous structure render SBA-15 mesoporous molecular sieves to serve as an attractive catalysts/catalyst carriers through improved mass transport diffusion during reactions (Agarwal et al., 2018). Up to now, various SBA-15-based catalysts, such as Fe-grafted SBA-15 (Nozaki et al., 2002), Co-substituted SBA-15 (Cui et al., 2010), single-site SBA-15 supported zirconium (Xu et al., 2015), molybdenum on SBA-15 (Azofra et al., 2018), and Ti-substituted mesoporous SBA-15 (Yu Y. et al., 2020), have been successfully developed. Meanwhile, the metal modified SBA-15 catalysts have been widely applied in various reactions, such as epoxidation, hydrogen generation, selective hydrogenation, dye degradation, ethanol oxidation, water splitting, soot combustion, and esterification. Especially, titanium-based catalysts such as TiO_2_/Nb_2_O_5·_nH_2_O and phosphated TiO_2_ (P-TiO_2_) have attracted much attention in the conversion of carbohydrates to HMF owing to their outstanding catalytic performance (DaVià et al., 2017; Rao et al., 2019; Huang et al., 2020). Zhang et al. (2019) used Al_2_O_3_-TiO_2_ Modified Sulfonated Carbon as a catalyst to catalyze the conversion of glucose dehydration to HMF and obtained 57.4% yield at 130°C. In those established catalytic systems, Ti^4+^ served as the Lewis acid sites for carbohydrates dehydration. Based on mesoporous SBA-15 with improved mass-transport diffusion and Ti^4+^ with strong Lewis acidity, we envision that mesoporous SBA-15 modified with Ti^4+^ will exhibit excellent catalytic performance for synthesis of HMF from fructose dehydration.

Herein, we demonstrated that Ti-SBA-15 solid acid prepared by a facile thermal synthesis method could facilitate the dehydration of fructose to produce HMF. Characteristic analysis of Ti-SBA-15 from XRD and TEM revealed that Ti groups were successfully doped into SBA-15 without the destruction of mesoporous structure. Meanwhile, the acidic properties (type, strength, and amount) of various Ti-SBA-15 samples were also investigated by pyridine-FTIR and NH_3_-TPD analysis. Various reaction parameters including reaction time, reaction temperature, and catalyst amount were investigated to explore the optimized reaction conditions. In particular, 100% fructose conversion with 82% HMF yield was obtained over Ti-SBA-15 (120) catalyst in DMSO under mild reaction conditions (140°C, 1 h). Furthermore, the catalytic performance of Ti-SBA-15 (120) in the fructose-to-HMF conversion was retained after fifth recycles, clearly suggesting its high stability.

## Materials and Methods

### Chemicals

Fructose (98%–102%) was purchased from the Sinopharm Chemical Reagent Co., Ltd. 5-Hydroxymethylfurfural (HMF, 97%), P123 (EO_20_PO_70_EO_20_), tetraethyl orthosilicate (SiO_2_ ≥ 28.4%), HCl (36%–38%), titanium (IV) chloride (98%), and dimethyl sulfoxide (DMSO, 99.5%) were purchased from Shanghai Titan Scientific Co., Ltd. All chemicals were used as supplied without any further purification. Besides, deionized water (DI water, 18.2 MΩ cm) used in this work was prepared using a water purification system (Heal Force, Shanghai Shengke Equipment Co., Ltd.).

### Preparation of Ti-SBA-15

The preparation procedure of Ti-SBA-15 was similar to the previously reported literature (Lin et al., 2018). In a typical process, 2 g of Pluronic P123 was dissolved in 60 ml of 2 mol/L hydrochloric acid and 15 ml H_2_O at 40°C under stirring for 12 h. Then, 11.2, 5.6, or 2.8 ml of titanium chloride (0.5 mol/L) was added followed by 4.2 ml of tetraethylorthosilicate to yield a Si/Ti of 30, 60, and 120. The mixture was stirred at 40°C for 5 h and subsequently hydrothermally treated at 100°C for another 24 h. The solid product was collected by filtration, washing several times with deionized water, and dried at 100°C overnight before calcined at 550°C in air for 6 h to remove the organic template. The resultant materials prepared with different amounts of titanium (IV) are denoted as Ti-SBA-15(x), where x represents the Si/Ti atomic ratio used in the reaction.

### Catalyst Characterization

The small-angle XRD diffraction patterns (0.5–5°) of as-prepared materials were collected with a Bruker D8 power diffractometer using Cu-Kα radiation (40 kV, 40 mA), at a scan rate of 0.5° min^−1^. TEM (Thermo Fisher Talos F200X) at 200 kV was used to examine the catalyst morphology. Nitrogen adsorption–desorption isotherms of as-prepared materials were measured on a Micro for TriStar II Plus 2.02 at −196°C. The samples were heated in a vacuum at 120°C for 6 h before the measurements to remove moisture and volatile impurities. On the basis of the desorption data, the surface area was calculated using the BET method while the Barrett–Joyner–Holanda (BJH) method was used to calculate the pore size distribution. To determine the total acidity of catalysts, the NH_3_-TPD (temperature programmed desorption) was performed in an AutoChemII analyzer. The sample tube was filled with 0.1 g of catalysts and heated at 350°C for 1 h in a helium environment. After cooling to 50°C，the samples were subjected to NH_3_/He (10%) mixed gas flow for 1 h. Then, the physisorbed NH_3_ was removed by changing the gas flow to helium for 1 h at 50°C. The TPD was then measured at a heating rate of 10°C min^−1^ (maximum desorption temperature was 550°C). Pyridine-IR data were collected using a PerkinElmer Frontier FT-IR spectrometer, with 64 scans at an effective resolution of 1 cm^−1^. A sample of 10 mg was pressed into a self-supporting wafer. The samples were pretreated at 350°C under vacuum prior to adsorption and then cooled to room temperature when pyridine vapor was introduced into the cell. At 150°C, the samples were heated in a vacuum, and the spectra were recorded at room temperature. The Ti content in the catalysts and filtrate after removal of Ti-SBA-15(120) was measured by ICP-OES.

### Catalytic Test

All fructose dehydration experiments were carried out in a 15-ml pressure tube with magnetic stirring. In a typical reaction, fructose (0.1 g) was mixed with Ti-SBA-15 at different mass ratios (1:1–20:1), followed by 10 ml of DMSO. The reactor was sealed and immersed in a heated at different temperatures (110–170°C) oil bath with stirring at a speed of 500 rpm for 0.5–5 h (Yu X et al., 2020). Time zero was established when the reactor was immersed in the oil bath. After the reaction, the liquid phases were passed through a 0.45-µm filter, and products in both aqueous and organic phases were analyzed by a high-performance liquid chromatography (HPLC).

HPLC analysis was performed using a Shimadzu liquid chromatography system fitted with a UV detector (SPD-16) and a Refractive Index (RI) detector (RID-20A), respectively. Fructose conversion was quantified with a Cosmosil packed column of D-sugars (4.6 mm, I.D. × 250 mm) and RI detector (RID-20A), using acetonitrile and water (3:1, v/v) as mobile phase at 30°C with a flow rate of 1 ml/min. HMF was monitored with a Cosmosil C18-AR-II packed column (4.6 mm, I.D × 150 mm), using a mobile phase consisting of methanol and water with phosphoric acid (20:80) at a flow rate of 1 ml/min, with a UV detector (SPD-16) and the column temperature was 30°C.

## Results and Discussion

### Catalyst Characterization of Ti-SBA-15 Catalysts

**FIGURE 2:**
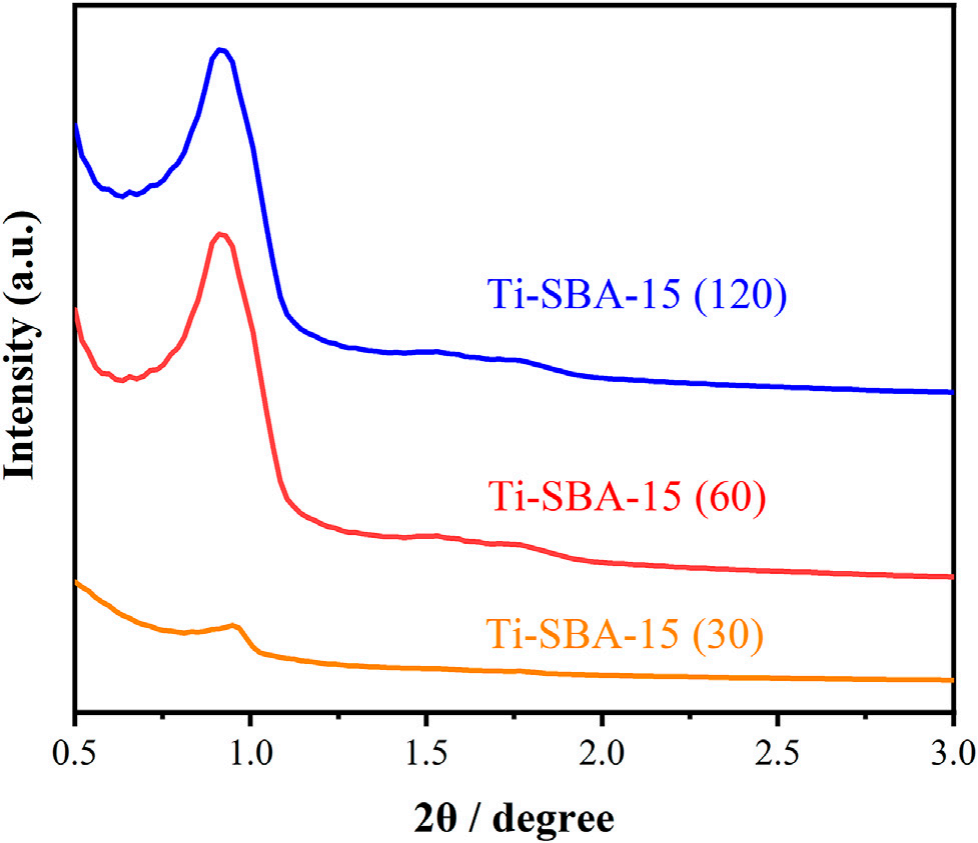
Powder x-ray diffraction of Ti-SBA-15 catalysts.

The low-angle XRD patterns of Ti-SBA-15(30), Ti-SBA-15(60), and Ti-SBA-15(120) catalysts are shown in Figure 2. The materials of Ti-SBA-15(60) and Ti-SBA-15(120) exhibit a very strong peak at about 2θ = 1.0°, which is attributed to the (100) plane of the SBA-15 material. Another two weak peaks between 1.5° and 2.0° are ascribed to the (110) and (200) planes, respectively. The results indicated that the Ti-SBA-15 catalysts possessed a two-dimensional hexagonal mesoporous structure with well ordered. The presence of those peaks confirms that the hexagonal mesoporous structure is also maintained after Ti group incorporation. In contrast, in the case of XRD patterns of Ti-SBA-15(30), a weak peak of the (100) plane is clearly observed while the peaks assigned to the (110) and (200) plane are not observed, indicating the partly collapsing of the mesoporous structure of SBA-15. The low-angle XRD patterns of Ti-SBA-15 catalysts showed that the intensity of all diffraction peaks gradually decreased with increasing Ti content, suggesting a decrease of long-distance order.

**FIGURE 3:**
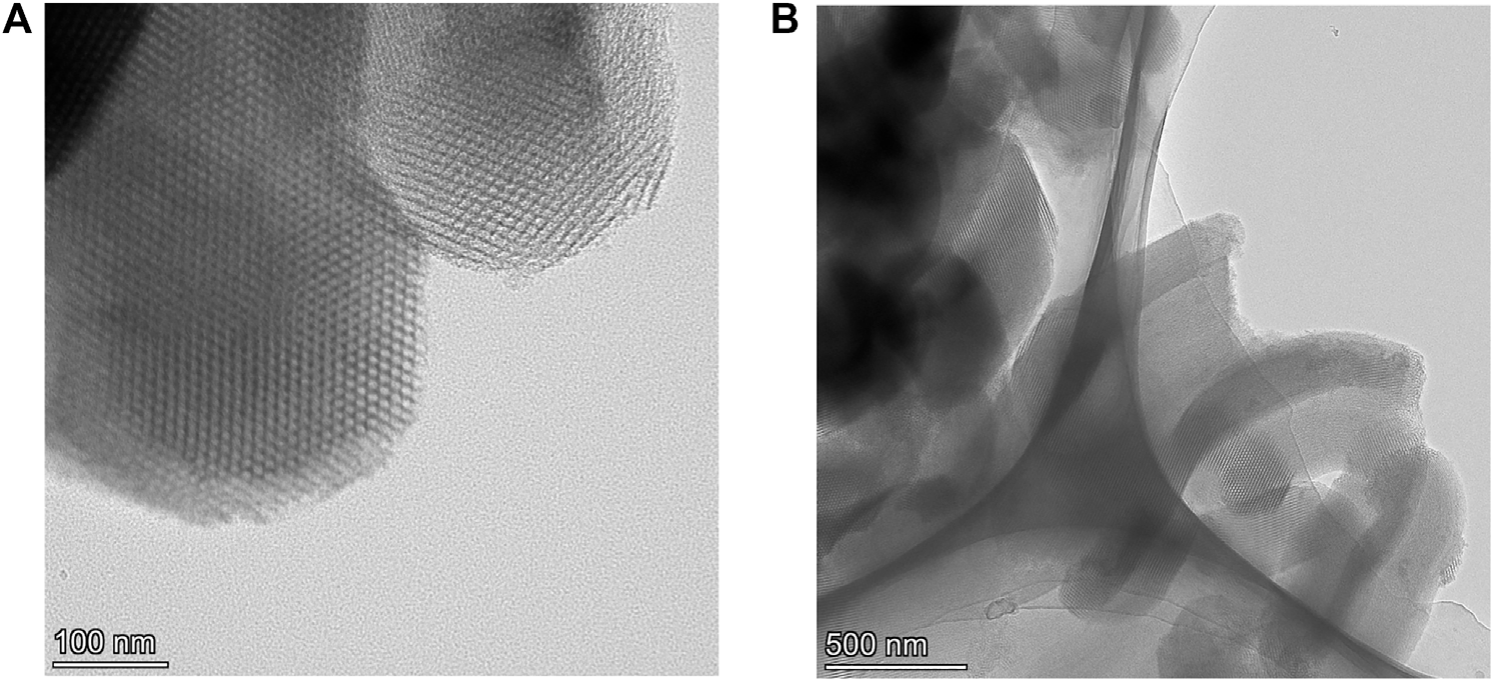
TEM images of Ti-SBA-15 (120).

The TEM images of Ti-SBA-15(120) are presented in Figure 3. It shows the highly ordered hexagonal arrangement of the channels and clearly demonstrates the retention of the periodic structure. The prepared Ti-SBA-15(120) sample preserved with one-dimensional channels, indicating a 2D hexagonal (p6mm) mesostructure, which is consistent with XRD results, also similar to those reported in the literature (Yu Y.et al., 2020).

**FIGURE 4:**
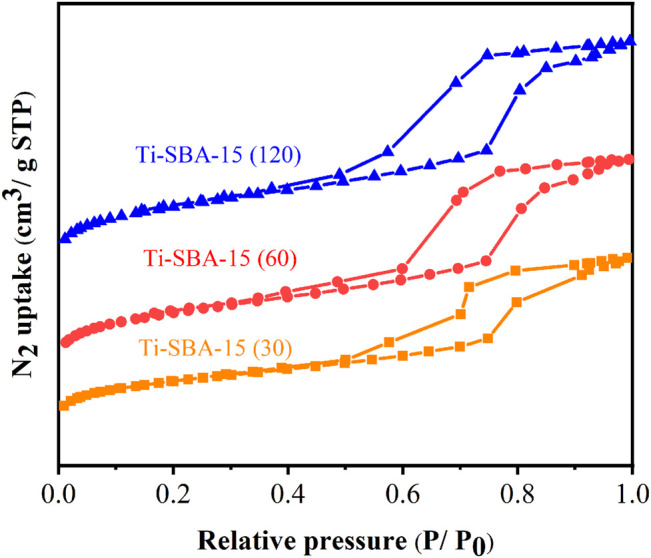
N_2_ adsorption–desorption isotherms of Ti-SBA-15 catalysts.

The textural properties of the Ti-SBA-15(30), Ti-SBA-15(60), and Ti-SBA-15(120) catalysts were studied by nitrogen physisorption at 77 K (Figure 4), and the detailed data about textural properties of all catalysts are summarized in Table 1. For all samples, the adsorption branch of the isotherms presented two adsorption phenomena, wherein the adsorption of nitrogen at low relative pressure (P/P_0_ < 0.1) mainly occurred by monolayer adsorption on micropores, while the samples showed a capillary condensation step at relative pressure at 0.6 < P/P_0_ < 0.8. The obtained results clearly indicate the presence of the mesopores within Ti-SBA-15 catalysts. The N_2_ adsorption–desorption curves of all Ti-SBA-15 catalysts displayed type IV isotherms, which is characteristic of typical mesoporous molecular sieves (Figure 4). This finding is also in well accordance with XRD spectrum analysis. As shown in Table 1, the BET surface area and total pore volume of the as-prepared materials significantly increased from 696 to 967 m^2^/g and 0.95–1.29 cm^3^/g, respectively, with the decrease of Ti content.

**TABLE 1:** Physio-chemical properties of catalysts.

Entry	Catalyst	BET surface area (m^2^ g^−1^)^a^	Pore volume (cm^3^ g^−1^)^a^	Pore size (nm)^a^	Ti content (wt%)^b^
1	Ti-SBA-15 (30)	696	0.95	5.50	16.56
2	Ti-SBA-15 (60)	937	1.22	5.21	3.11
3	Ti-SBA-15 (120)	967	1.29	5.36	0.16

a BET, surface area, pore volume, and pore size were measured by N_2_-adsorption–desorption.

b Ti content was determined by ICP-OES.

**FIGURE 5:**
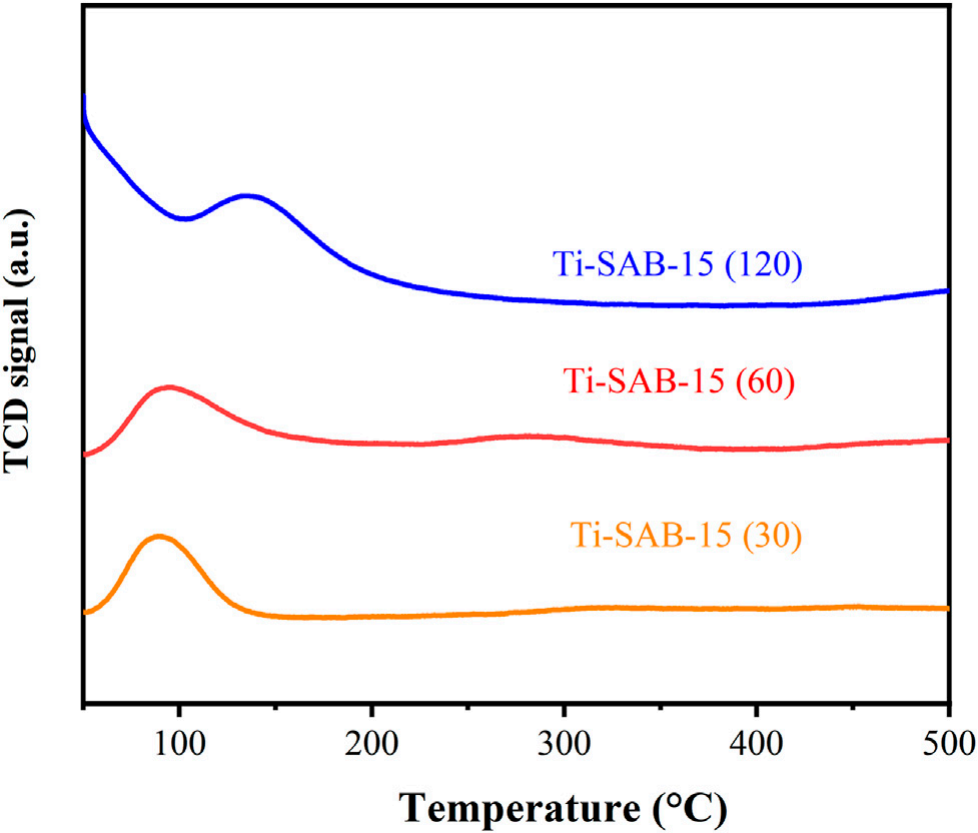
NH_3_-TPD curves of different Ti-SBA-15 catalysts.

The acid concentration of the Ti-SBA-15 samples was determined by temperature-programmed desorption of ammonia (Figure 5 and Table 2, entries 1–5). It is clear that all of the Ti-SBA-15 catalysts mainly possessed weak (<300°C) and moderate acid sites (300–500°C), and the acid density of all catalysts increases with the decrease of Ti content. It is also worth noting that Ti-SBA-15(120) has the highest concentration of acid sites (1.32 mmol/g) than the others. The Ti-SBA-15(120) catalyst has higher weak acidity than other catalysts. The weak acid amount of Ti-SBA-15 samples decreased with decreasing the Si/Ti ratio (or increasing titanium contents) from 120 to 30. As more Ti is introduced, the proportion of medium acidic sites gradually increases, while the proportion of weak acidic sites gradually decreases, which indicates that Ti is introduced as a moderate acidic site. The Ti content of Ti-SBA-15(30), Ti-SBA-15(60), and Ti-SBA-15(120) was 16.56%, 3.11%, and 0.16%, respectively (Table 1). The results show that the measured values inevitably deviate from the calculated values, indicating that not all Ti species enter the molecular sieves.

**TABLE 2:** Acid strength, type, and density of Ti-SBA-15.

Entry	Catalyst	Acid strength distribution (%) ^a^		Total acid density (mmol/g) ^a^	L/(L+B) ^b^
		<300°C	300–500°C		
1	Ti-SBA-15 (30)	58.5	41.5	0.53	0.92
2	Ti-SBA-15 (60)	69.6	30.4	0.70	0.868
3	Ti-SBA-15 (120)	100	0	1.32	1

a Acid strength distribution and total acid density were measured by NH_3_-TPD.

b L/(L + B) was determined by Pyridine-IR.

**FIGURE 6:**
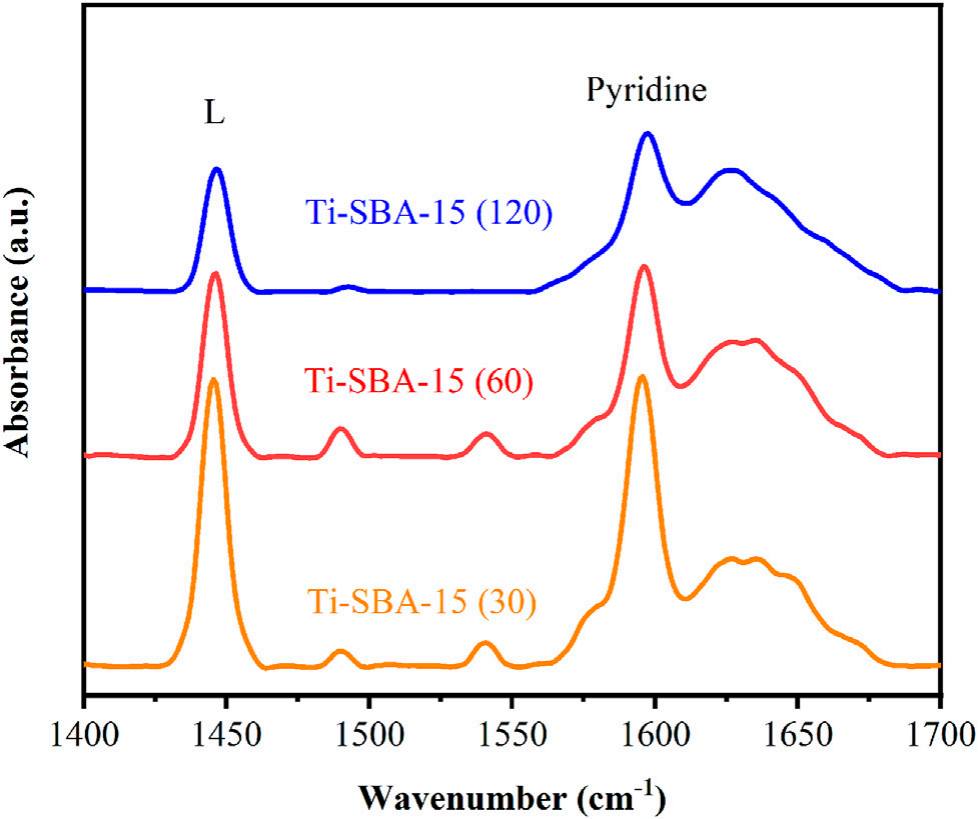
Pyridine FTIR spectra of Ti-SBA-15 samples collected after treatment at 150°C under vacuum.

To learn more about the acidic nature of Ti-SBA-15 catalysts, the pyridine-FTIR spectra were collected as shown in Figure 6. The band at 1,590 cm^−1^ in Figure 6 corresponds to the physical adsorption of weak Lewis acid sites (Ravi et al., 2020). The characteristic bands at 1,450 and 1,623 cm^−1^ belonging to pyridine bounded to the strong Lewis acid sites are observed in all Ti-SBA-15 samples, demonstrating that the as-prepared Ti-SBA-15 catalysts possessed Lewis acid sites (Yu Y.et al., 2020). The bands at 1,545 cm^−1^ with low intensity corresponding to Brönsted acid sites were also clearly observed for Ti-SBA-15(60) and Ti-SBA-15(30) catalysts, suggesting the presence of a small amount of Brönsted acid sites within the Ti-SBA-15(30) and Ti-SBA-15(60) materials. Furthermore, the band at 1,490 cm^−1^ attributed to pyridine adsorption on both Lewis and Brönsted acid sites were also observed for all Ti-SBA-15 samples as exhibited in Figure 6. These results showed that Ti-SBA-15(60) and Ti-SBA-15(30) samples had a large amount of Lewis acid sites along with a small amount of Brönsted acid sites. For as-prepared Ti-SBA-15 samples, the TiO_4_ units of the hydrated surface titanium species on SBA-15 might be connected by Ti–O–Si bonds (Sudarsanam et al., 2020). As the structure of surface titanium species is reversible during hydration and dehydration, these titanium species possess little Brönsted acidity (Peng et al., 2017). The absence of Brönsted acid sites in Ti-SBA-15(120) is due to the low content of titanium (Table 2, entries 6).

### Equation Dehydration of Fructose Into HMF Over Different Catalysts

The catalytic performances of Ti-SBA-15 catalysts with different Si/Ti ratios and without catalyst in catalyzing dehydration of fructose are summarized in Table 3. Only 57% yield of HMF was obtained in the blank experiment, far inferior to HMF yield (82%) obtained with the Ti-SBA-15(120) catalyst, which clearly demonstrated the high activity of the Ti-SBA-15(120) catalyst. All the catalysts demonstrated high conversion rate to fructose (100%) under the reaction conditions. As the amount of Ti in the catalyst increased, the reactivity diminished for fructose dehydration. The highest HMF yield (82%) was obtained over Ti-SBA-15(120). The yield from the different catalysts did not vary greatly for 1 h in our study. In order to find the activity difference among the as-prepared catalysts, we have lowered the reaction temperature and shortened the reaction time. Note that high temperature with a short time (130°C for 20 min) showed a significant difference in HMF yield. As shown in the Supplementary Table S2, The Ti-SBA-15(120) catalyst gave the greatest HMF yield of about 9%. There was almost no HMF production when the experiment was carried out in the presence of Ti-SBA-15(30) or Ti-SBA-15(60) catalysts (only 1 and 2%). It was observed that Ti-SBA-15(120) showed the highest activity among the different catalysts employed in this reaction. The excellent catalytic performance of Ti-SBA-15(120) was associated to its high surface area, large pore size, and high pore volume. Therefore, we chose the Ti-SBA-15(120) catalyst for the following investigation.

**TABLE 3:** Influence of Si/Ti ratio on fructose conversion, and HMF yield over Ti-SBA-15 catalysts (reaction conditions: 0.1 g fructose, 0.01 g catalysts, 140°C, 1 h, 10 ml DMSO).

Catalyst	Fructose conversion (%)	HMF yield (%)
Without catalyst	100	57
Ti-SBA-15 (30)	100	76
Ti-SBA-15 (60)	100	79
Ti-SBA-15 (120)	100	82

### Effect of Reaction Time, Temperature, and Amount of Catalyst

**FIGURE 7:**
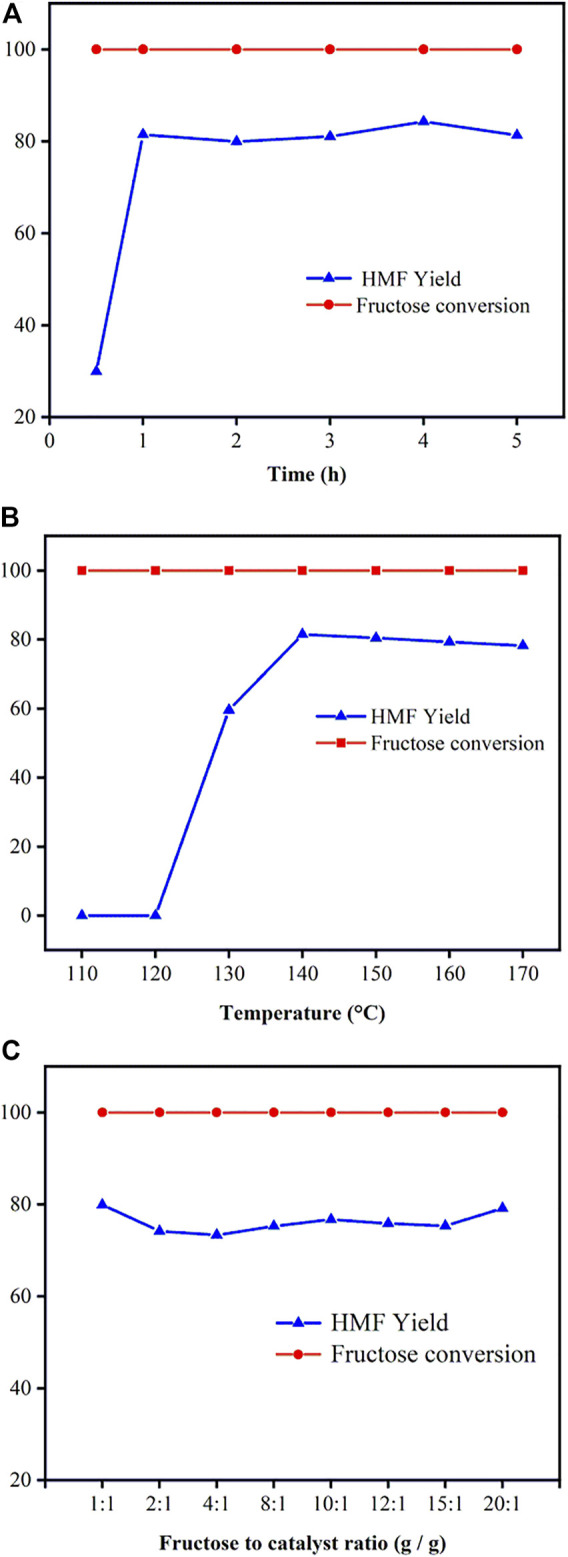
Dependence of fructose (0.1 g) conversion yield and yield for HMF **(A)** effect of reaction time; **(B)** effect of reaction temperature; **(C)** effect of catalyst dosage [reaction conditions: **(A)** 0.1 g fructose, 0.01 g catalysts, 140°C, 10 ml DMSO; **(B)** 0.1 g fructose, 0.01 g catalysts, 1 h, 10 ml DMSO; **(C)** 0.1 g fructose, 140°C, 1 h, 10 ml DMSO].

In accordance with the initial catalysts screening above, Ti-SBA-15(120) was the most effective catalyst for fructose dehydration reaction. Thus, the effect of the reaction time and temperature on fructose-to-HMF conversion was examined with Ti-SBA-15(120) as catalysts and the results are shown in Figure 7. Figure 7A shows that the fructose was almost completely consumed at 140°C after 30 min. The yield of HMF increased remarkably with the reaction time from 0.5 to 1 h. However, the yield of HMF showed a decreased trend when the time was extended to 5 h owing to the production of by-products (e.g., humins and levulinic acid). So, the optimal reaction time was chosen as 1 h.

The effect of reaction temperature (110–170°C) on dehydration of fructose into HMF over Ti-SBA-15(120) catalyst was examined as shown in Figure 7B. Obviously, the yield of HMF was strongly influenced by the reaction temperature. For example, the yield of HMF increased with the increase of reaction temperature, and an 82% yield of HMF with 100% fructose conversion was acquired at 140°C after 1 h. At a higher temperature (150, 160, and 170°C), the yield of HMF was lower than that obtained at 140°C. Moreover, the high temperature results in more by-products derived from further degradation of HMF (Zhao et al., 2011; Wang et al., 2019). The optimal reaction temperature and reaction time were thus 140°C and 1 h, respectively.

The catalyst dosage is also an important factor affecting hydrolysis processes, so it is necessary to explore the effect of Ti-SBA-15(120) dosage on the dehydration of fructose. The effect of Ti-SBA-15 dosage on the dehydration of fructose over the Ti-SBA-15(120) catalyst is shown in Figure 7C. When the Ti-SBA-15(120) dosage is 0.005 g (mass ratio of fructose/catalyst is 20:1), the Lewis acid sites were already saturated for the hydrolysis of fructose. Further increase in the amount of Ti-SBA-15 dosage from 0.005 to 0.1 g (mass ratio of fructose/catalyst from 20:1 to 1:1) showed no significant increase in the HMF yield, which may be attributed to the formation of the side products (e.g., humins), which probably stemmed from polymerization of HMF in the presence of a high amount of the catalyst loading (Girisuta et al., 2006). So, the optimal mass ratio for fructose and Ti-SBA-15 is 20:1.

### Catalyst Stability

**FIGURE 8:**
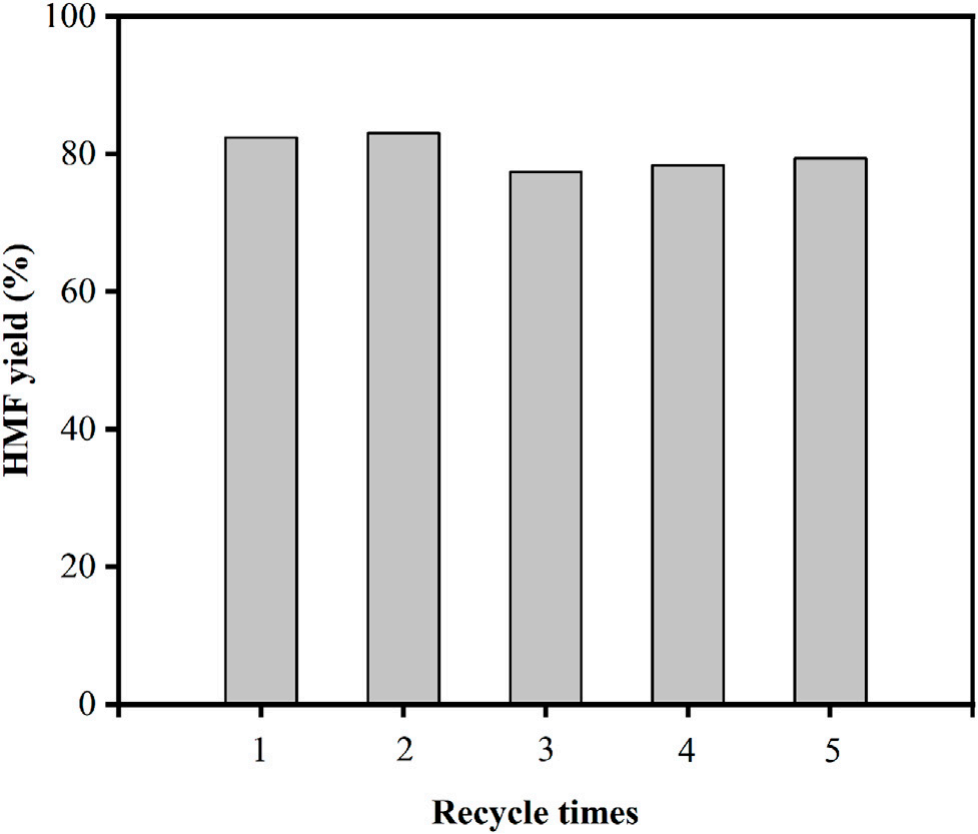
Cycle use of Ti-SBA-15 (120) catalyst for fructose conversion reaction (reaction conditions: 0.1 g fructose, 0.01 g catalysts, 10 ml DMSO, 140°C, 1 h).

Heterogeneous catalysts with high stability are critical for the advancement of aqueous phase biomass processing. The catalytic stability of the Ti-SBA-15(120) catalyst was investigated in the conversion of fructose to HMF in 10 ml of DMSO (140°C, 1 h). After the reaction was completed, the reused catalyst was collected by centrifugation, washed three times with DMSO and acetone in ultrasonic (5 min), dried at 100°C for 2 h, and then calcined at 550°C for 3 h, before being used for the next run under the same reaction conditions. Ti-SBA-15(120) recycle test results are shown in Figure 8. The HMF yield remained at 80% in the fifth cycle, indicating that the catalyst can be recycled effectively without losing catalytic activity, strongly demonstrating the high stability of the Ti-SBA-15(120) catalyst for fructose dehydration.

In addition, XRD patterns and N_2_ adsorption–desorption isotherms of fresh and used Ti-SBA-15(120) catalysts have been compared as shown in Supplementary Figures S1, S2. N_2_ adsorption–desorption analysis shows that there is a slight discrepancy in BET surface area (967–735 m^2^/g) and pore volume (1.29–1.07 cm^3^/g) between fresh and used Ti-SBA-15(120) (Supplementary Table S1). The used Ti-SBA-15(120) catalyst kept its original mesoporous structure (Supplementary Figure S1). The reusability experiments have shown that Ti-SBA-15(120) was a stable and efficient catalyst for the synthesis of HMF. Furthermore, we also determined the Ti concentration in the filtrate after removing the catalyst using ICP-OES. The Ti species leaching during the reaction was extremely minimal (<0.00002%); thus, the active species leaching during the reaction is negligible. These results further proved the good stability of Ti-SBA-15(120) in the hydrolysis of fructose to HMF.

## Conclusion

The Ti-SBA-15 catalyst that we developed is an efficient catalyst for the production of HMF from fructose. Characterization results revealed that the addition of Ti into SBA-15 materials had no obvious influence on its pristine mesoporous structure. The Ti-SBA-15 also had a high surface area, large pore size, and high pore volume. Ti-SBA-15(120) proved to be highly active to HMF, and a high yield of HMF of 82% was obtained by the hydrolysis of fructose in DMSO. Consecutive use of Ti-SBA-15(120) demonstrated that, after the fifth cycle, the activity loss is not significant in the conversion of fructose-based substrates to HMF. Ti-SBA-15 exhibited better activity and stability.

## Data Availability

The original contributions presented in the study are included in the article/Supplementary Material, further inquiries can be directed to the corresponding author.
