# Supplementary material for: Effective Dehydration of Fructose Over Stable Ti-Doped SBA-15 Catalysts
Source: Front Chem. 2022 Jan 5;9:817417. doi: 10.3389/fchem.2021.817417 (PMC8769218; doi:10.3389/fchem.2021.817417)
Supplement: Supplementary file 1 [file DataSheet1.DOCX]

Supplementary Material

**Supplementary Table** **S1** Physio-chemical properties of Ti-SBA-15 (120) and reused Ti-SBA-15 (120) catalysts.

| **Entry** | **Catalyst** | **BET surface area**  **(m^2^ g^-1^) ^1^** | **Pore volume**  **(cm^3^ g^-1^) ^1^** | **Pore size**  **(nm) ^1^** |
| --- | --- | --- | --- | --- |
| 1 | Ti-SBA-15 (120) | 967 | 1.29 | 5.36 |
| 2 | Reused Ti-SBA-15 (120) | 735 | 1.07 | 5.85 |

**

**

**Supplementary Figure 1.** Powder X-ray diffraction of Ti-SBA-15 (120) and reused Ti-SBA-15 (120) catalysts.





**Supplementary Figure 2.** N_2_ adsorption-desorption isotherms of Ti-SBA-15 (120) and reused Ti-SBA-15 (120) catalysts.

**Supplementary Table** **S2** Influence of Si/Ti ratio on HMF yield over Ti-SBA-15 catalysts. (Reaction condition：0.1 g fructose, 0.01 g catalysts, 130 °C, 20 min, 10 mL DMSO)

| **Entry** | **Catalyst** | **HMF yield (%)** |
| --- | --- | --- |
| 1 | Ti-SBA-15 (30) | 1 |
| 2 | Ti-SBA-15 (60) | 2 |
| 3 | Ti-SBA-15 (120) | 9 |
